# Hypolipidemic Effects of Beetroot Juice in SHR-CRP and HHTg Rat Models of Metabolic Syndrome: Analysis of Hepatic Proteome

**DOI:** 10.3390/metabo13020192

**Published:** 2023-01-28

**Authors:** Jan Šilhavý, Petr Mlejnek, Miroslava Šimáková, Hana Malínská, Irena Marková, Martina Hüttl, Denisa Miklánková, Ludmila Kazdová, Marek Vrbacký, Alena Pecinová, Tomáš Mráček, Michal Pravenec

**Affiliations:** 1 Institute of Physiology, Czech Academy of Sciences, 14200 Prague, Czech Republic; 2 Centre for Experimental Medicine, Institute for Clinical and Experimental Medicine, 14021 Prague, Czech Republic

**Keywords:** spontaneously hypertensive rat, hereditary hypertriglyceridemic rat, beetroot, lipids, proteomics, glycerophospholipid metabolism, mTOR signalling

## Abstract

Recently, red beetroot has attracted attention as a health-promoting functional food. Studies have shown that beetroot administration can reduce blood pressure and ameliorate parameters of glucose and lipid metabolism; however, mechanisms underlying these beneficial effects of beetroot are not yet fully understood. In the current study, we analysed the effects of beetroot on parameters of glucose and lipid metabolism in two models of metabolic syndrome: (i) transgenic spontaneously hypertensive rats expressing human C-reactive protein (SHR-CRP rats), and (ii) hereditary hypertriglyceridemic (HHTg) rats. Treatment with beetroot juice for 4 weeks was, in both models, associated with amelioration of oxidative stress, reduced circulating lipids, smaller visceral fat depots, and lower ectopic fat accumulation in the liver compared to the respective untreated controls. On the other hand, beetroot treatment had no significant effects on the sensitivity of the muscle and adipose tissue to insulin action in either model. Analyses of hepatic proteome revealed significantly deregulated proteins involved in glycerophospholipid metabolism, mTOR signalling, inflammation, and cytoskeleton rearrangement.

## 1. Introduction

Ageing and accumulation of visceral adipose tissue are associated with an increased risk of cardiovascular and metabolic complications, such as hypertension and type 2 diabetes. Decreased bioavailability or synthesis of nitric oxide (NO) from endothelial NO synthase (eNOS) has been proposed as a common underlying molecular mechanism that leads to predisposition to ageing-dependent disorders [1]. Endothelial dysfunction, which is often associated with oxidative stress, may link metabolic and cardiovascular disease in humans, and new strategies to restore NO bioavailability may have an important therapeutic role [2]. An additional source of NO, independent of eNOS activity, is represented by dietary nitrate from some leafy and root vegetables. In addition to inorganic nitrates, beetroot also contains ingredients, such as polyphenols, pigments (betalains), and organic acids, that may also ameliorate metabolic and hemodynamic disturbances [3,4]. Multiple biological effects of beetroot or extracted compounds were reported to include antioxidative effects [5,6], activation of the *Nrf2* transcription factor [7], reduced inflammatory cytokines (TNF-α, IL-6, IL-10) [8], prevention of salt-sensitive hypertension in rats [9], reduced blood pressure in humans with essential hypertension [10,11], lipid-lowering effects in both rats [12,13,14] and humans [15,16], and antidiabetic effects, mainly in rodent models [17,18,19]. However, the mechanisms responsible for the beneficial effects of beetroot are not fully understood.

In the current study, we tested the effects of beetroot juice on metabolic parameters in animal models of inflammation, dyslipidemia, and insulin resistance, in the spontaneously hypertensive rat with transgenic expression of human C-reactive protein (SHR-CRP rats) and in hereditary hypertriglyceridemic (HHTg) rats. To search for the molecular mechanisms of anti-inflammatory, antioxidative, antidiabetic, and hypolipidemic effects of beetroot, we performed an analysis of the hepatic proteome. We tested the hypothesis that categorising groups of proteins according to the expression patterns they show across experimental conditions will provide clues regarding the function of novel proteins based on both the expression categories to which they belong and the functions of known proteins that fall within those same categories. The identification of such proteins will, in turn, generate a whole new set of questions pertaining to their potential roles in disease pathogenesis, prevention, and management.

## 2. Experimental Design

### 2.1. Animals

**Figure 1:**
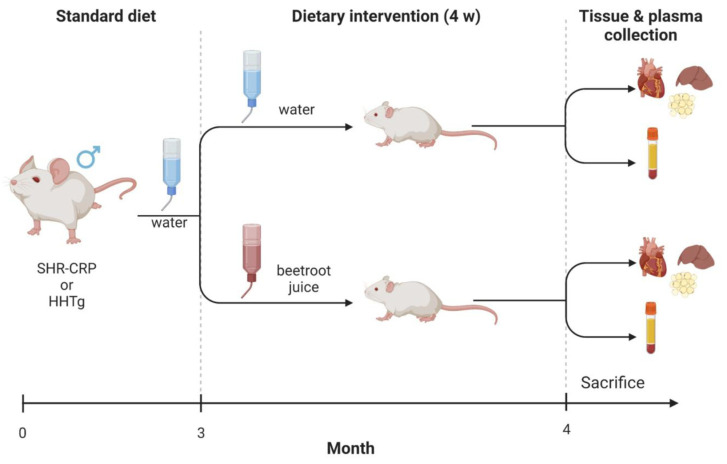
The protocol timeline. Male SHR-CRP and HHTg rats were fed standard laboratory chow and tap water ad libitum. At the age of 3 months, animals from each strain were divided into 2 groups: the experimental groups drank ad libitum beetroot juice for 4 weeks, while their respective controls drank tap water.

Transgenic SHRs (hereafter referred to as SHR-CRPs) were derived by microinjections of fertilised ova with a construct containing the cDNA for human CRP, under control of the apoE promoter, with the objective of driving expression of the CRP transgene in the liver, where CRP is normally produced [20]. HHTg (hereditary hypertriglyceridemic) rats represented a prediabetic model that was characterised by genetically determined hypertriglyceridemia, insulin resistance in peripheral tissues, low-grade inflammation in the absence of obesity, and fasting hyperglycaemia [21,22]. The SHR-CRP rats were provided by the Institute of Physiology, Czech Academy of Sciences, Prague, Czech Republic, and the HHTg rats were obtained from the Institute for Clinical and Experimental Medicine, Prague, Czech Republic. Three-month-old male SHR-CRP and HHTg rats were fed standard laboratory chow ad libitum, and animals from each strain were divided into 2 groups. The experimental groups (*n* = 6 per group) were treated for 4 weeks with ad libitum drinking solution that was prepared by mixing 920 mL of tap water with 80 mL of organic beetroot juice, which had a nitrate concentration of 1.24 mg/mL (organic beetroot juice, James White Drinks Ltd., Ipswich, UK) (Figure 1). The nitrite concentration of the beetroot juice was <0.002 mg/mL. The nitrate and nitrite concentrations in the beetroot juice were determined by independent testing with an isotachophoresis method at the University of Chemistry and Technology, Faculty of Food and Biochemical Technology, Prague. Control groups (*n* = 6 per group) received tap water ad libitum for drinking. According to testing conducted by the Prague water supply authority, the background concentration of nitrate was approximately 0.0201 mg/mL, and the background concentration of nitrite was approximately 0.00001 mg/mL [9]. All rats were housed in an air-conditioned animal facility.

### 2.2. Biochemical Analyses

Rat serum CRP and human serum CRP were measured using ELISA kits (Alpha Diagnostics International, San Antonio, TX, USA). Serum levels of glucose, triglycerides, and both total and HDL cholesterol were measured using commercially available kits (Erba-Lachema, Brno, Czech Republic). NEFA levels were determined using an acyl-CoA oxidase-based colorimetric kit (Roche Diagnostics GmbH, Mannheim, Germany). Serum insulin concentrations were determined using a rat insulin ELISA kit (Mercodia, Uppsala, Sweden). Inflammatory parameters in serum MCP-1, IL-6, TNF-α, and leptin were measured by rat ELISA kits (eBioscience, Vienna, Austria; MyBioSource, San Diego, CA, USA; BioVendor, Brno, Czech Republic).

### 2.3. Measurement of Plasma and Tissue cGMP Concentrations

All analyses were performed with acetylation to achieve maximum sensitivity. Blood samples were immediately centrifuged at 4 °C and processed. Frozen tissue samples were crushed to a fine powder in liquid nitrogen, homogenised in 0.1 M HCl, and centrifuged at 4 °C. Levels of cGMP were measured in acetylated supernatant using a radioimmunoassay kit (IBL International GmbH, Hamburg, Germany).

### 2.4. Tissue Triglyceride and Cholesterol Measurements

To determine concentrations of triglycerides and cholesterol in the liver, muscle, and heart, tissue samples were powdered under liquid N_2_ and extracted in a mixture of chloroform:methanol at room temperature, after which 2% KH_2_PO_4_ was added and the solution was centrifuged. The organic phase was removed and evaporated under N_2_. The resulting pellet was dissolved in isopropyl alcohol, and triglyceride and cholesterol content were determined by enzymatic assay (Erba-Lachema, Brno, Czech Republic).

### 2.5. Parameters of Insulin Sensitivity in Skeletal Muscle and Adipose Tissue

Tissue insulin sensitivity was measured according to insulin-stimulated incorporation of glucose into skeletal muscle glycogen or visceral adipose tissue lipids. Diaphragm or epididymal adipose tissue was incubated for 2 h in 95% O_2_ with 5% CO_2_ in Krebs–Ringer bicarbonate buffer (pH 7.4) containing 0.1 μCi/mL of ^14^C-U glucose, 5 mmol/L of unlabelled glucose, and 2.5 mg/mL of bovine serum albumin (Fraction V, Sigma, Czech Republic), with or without 250 μU/mL of insulin. Glycogen and lipids were extracted, and incorporation of glucose into glycogen or lipids was determined by scintillation counting, as previously described [23]. Glucose oxidation was determined ex vivo in the isolated m. soleus by measuring the incorporation of ^14^C-U glucose into CO_2_, as previously described [23].

### 2.6. Parameters of Oxidative Stress

The activity of antioxidative enzymes and concentrations of lipoperoxidation products were measured as previously described [24]. The activity levels of antioxidant enzymes superoxide dismutase (SOD), glutathione peroxidase (GSH-Px), and glutathione reductase (GR) were analysed using Cayman Chemicals assay kits (Ann Arbor, MI, USA). Catalase (CAT) activity measurement was based on the ability of H_2_O_2_ to produce, along with ammonium molybdate, a colour complex that can be detected spectrophotometrically. Concentrations of conjugated dienes were determined by extraction in media (heptane:isopropanol 2:1) and measured spectrophotometrically in the heptane layer. MDA, a parameter of lipoperoxidation, was determined by assaying the reaction with thiobarbituric acid by the HPLC method with fluorescence detection. The levels of reduced (GSH) and oxidised (GSSG) forms of glutatione were determined by the HPLC method with fluorescence detection, using an HPLC diagnostic kit (Chromsystems, Gräfelfing, Germany).

### 2.7. Proteomic Analysis

Liver samples were pulverised in liquid nitrogen, solubilised in 1% SDS, and processed according to the SP4 no glass bead protocol [25]. About 500 ng of tryptic peptides were separated on a 50 cm C18 column using 2.5 h elution gradient, and were then analysed in a DDA mode on an Orbitrap Exploris 480 (Thermo Fisher Scientific) mass spectrometer equipped with a FAIMS unit. The resulting raw files were processed in MaxQuant v2.1.4.0. [26] with the label-free quantification (LFQ) algorithm MaxLFQ [27] using the rat proteome database (UP000002494_10116.fasta, UniProt Release 2022_01). Downstream analysis was performed in Perseus (v. 2.0.7.0) [28], and visualisation in GraphPad Prism 9. The mass spectrometry proteomics data have been deposited to the ProteomeXchange Consortium via the PRIDE partner repository with the dataset identifier PXD039605.

### 2.8. Statistical Analysis

All data are expressed as means ± S.E.M. We used the *t*-test or rank-sum test for two-group comparisons. Normality of distribution was tested by the Shapiro–Wilk method. Statistical significance was defined as *p* < 0.05. Functional analysis of the Kyoto Encyclopedia of Genes and Genomes (KEGG) pathways was performed with DAVID 2021 online software [29].

## 3. Results

### 3.1. The Effects of Beetroot Juice on Parameters of Glucose and Lipid Metabolism

**Figure 2:**
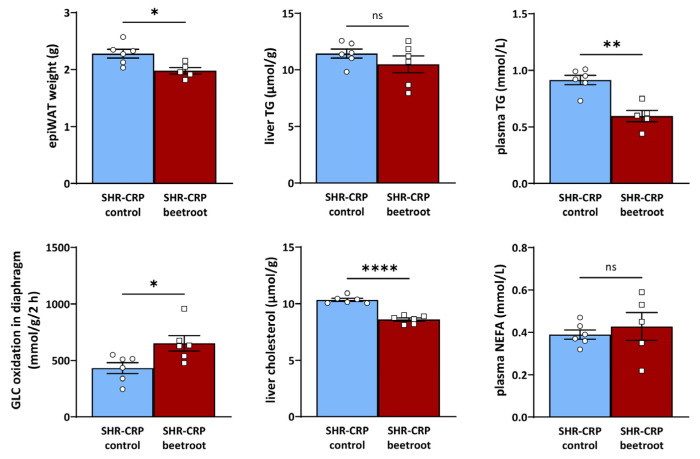
Parameters of lipid and glucose metabolism in SHR-CRP rats treated with beetroot and their untreated controls. *, **, and **** denote *p* < 0.05, *p* < 0.005, and *p* < 0.00005.

**Figure 3:**
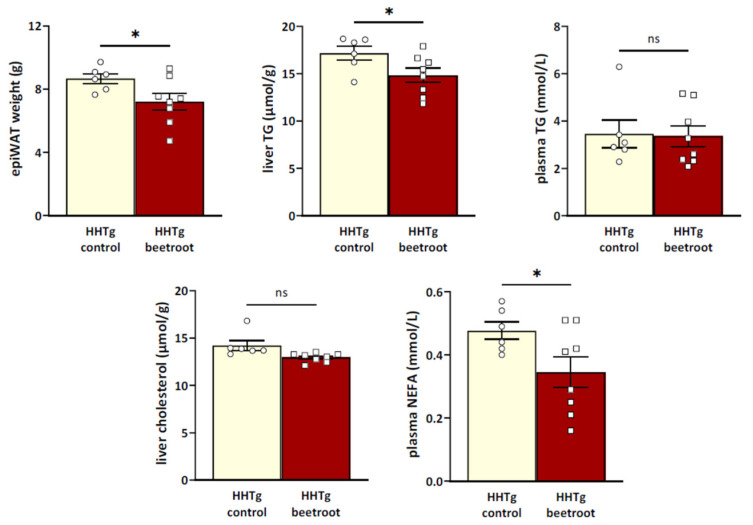
Parameters of lipid metabolism in HHTg rats treated with beetroot and their untreated controls. * denotes *p* < 0.05.

As can be seen in Table 1 and Figure 2 and Figure 3, beetroot juice-treated SHR-CRP and HHTg rats, when compared to their respective controls, showed similar body weights, but significantly reduced epididymal fat weights. In addition, SHR-CRP rats treated with beetroot juice showed lower concentrations of serum triglycerides, and HHTg beetroot-treated rats had lower serum NEFA levels when compared to the untreated controls (Figure 2 and Figure 3). Furthermore, beetroot juice treatment reduced hepatic cholesterol concentration in both SHR-CRP and HHTg rats and markedly reduced hepatic triglycerides in HHTg rats when compared to the untreated controls (Figure 2 and Figure 3). No significant changes in non-fasting glucose and insulin, nor in insulin action on glycogenesis in skeletal muscle or lipogenesis in epididymal fat, were observed (Table 1). On the other hand, SHR-CRP beetroot-treated rats showed significantly increased glucose oxidation in the diaphragm when compared to controls (Figure 2). Administration of beetroot juice had no effect on food and water intake (data not shown).

### 3.2. The Effects of Beetroot Juice on cGMP in Serum and Tissues

SHR-CRP rats treated with beetroot, when compared to the untreated controls, showed increased cGMP concentrations in serum and in myocardium (Table 1), suggesting better NO bioavailability.

### 3.3. The Effects of Beetroot Juice on Inflammatory and Oxidative Stress Parameters

As can be seen in Table 2, beetroot juice administration had no effects on inflammatory markers in either SHR-CRP and HHTg rats when compared to untreated controls. On the other hand, beetroot juice treatment of SHR-CRP rats was associated with reduced oxidative stress in the liver, the concentration of conjugated dienes was reduced, and catalase activity increased (Table 3). Beetroot treatment was associated with amelioration of oxidative stress in HHTg rats when the activity levels of the antioxidant enzymes SOD, GSH-Px, and CAT were significantly increased compared to the controls. However, no significant changes were detected in malondialdehyde (MDA) lipoperoxidation in either model (Table 3).

### 3.4. Analysis of Hepatic Proteome

**Figure 4:**
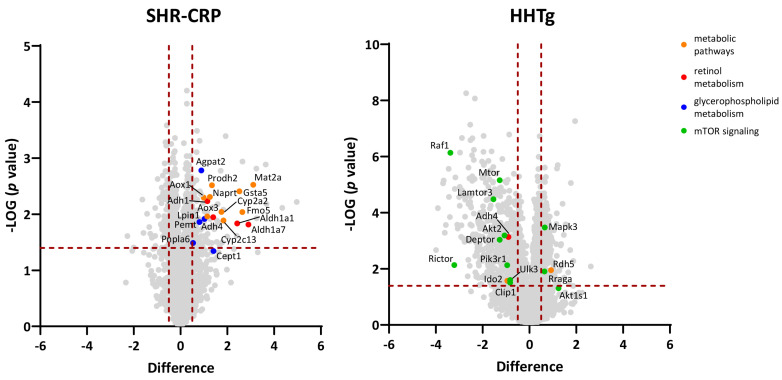
Proteomic analysis of liver samples. Label-free quantification—mass spectrometry analysis (LFQ-MS) of liver tissue from SHR-CRP and HHTg rats. Volcano plots depicting differential content of proteins upon treatment with beetroot juice are shown. The *X*-axis is the Log2 of linear fold change (beetroot–controls) and the *Y*-axis is the negative Log10 of the Benjamini–Hochberg corrected *t*-test *p* value. Vertical red dashed lines denote the cut-off for a linear fold change of 0.5 in either direction; horizontal red dashed line denotes a cut-off of 0.05 for *p* value. Proteins which belong to significantly changed KEGG pathways (see Table 4 and Table 5) are highlighted.

To search for the molecular mechanisms responsible for beetroot’s beneficial effects on metabolic parameters, we performed analysis of the hepatic proteome in SHR-CRP and HHTg rats that were treated with beetroot versus their untreated SHR-CRP and HHTg controls (Table S1). Figure 4 shows differentially expressed proteins and the metabolic pathways in which these proteins are involved. In SHR-CRP rats, beetroot treatment was associated with significant upregulation in the abundance of proteins from the KEGG metabolic pathways and overlapping retinol metabolism, steroid hormone biosynthesis, tyrosine metabolism, glycerophospholipid metabolism, and glutathione metabolism KEGG pathways (Table 4). Some of these proteins were implicated in regulation ectopic fat accumulation in the liver. In HHTg rats, beetroot treatment was associated with significant downregulation in the expression of proteins from mTOR signalling, Salmonella infection, and Alzheimer’s disease KEGG pathways (Table 5). Some of these proteins have been reported to regulate lipid metabolism in the liver, and to have intracellular trafficking and inflammation properties.

## 4. Discussion

In the current study, we analysed the effects of beetroot juice on metabolic syndrome in two animal models. In the SHR-CRP rats, disturbances in glucose and lipid metabolism were secondary to inflammation associated with transgenic expression of human C-reactive protein [20], while insulin resistance and hepatic steatosis in HHTg rats were secondary to hereditary hypertriglyceridemia [21,22]. The effects of beetroot on metabolic, inflammatory, and oxidative stress parameters were similar in both models, specifically included reduced weight of visceral adipose tissue and ectopic fat accumulation in the liver, as well as decreased concentrations of circulating lipids and the absence of insulin-sensitising effects in muscle and adipose tissue. Amelioration of oxidative stress in SHR-CRP rats was associated with increased concentrations of cGMP, which suggests greater availability of NO and NO-cGMP signalling.

Multiple studies, both in humans and in rodent models, have demonstrated significant anti-inflammatory, antioxidative, antidiabetic, hypolipidemic, and antihypertensive effects of beetroot treatment, as summarised by Hadipour et al. [3]. However, the mechanisms underlying these beneficial effects of beetroot are not fully understood. To search for molecular mechanisms which could be responsible for beetroot’s beneficial effects we analysed the hepatic proteome in both rat models. Hypolipidemic effects in SHR-CRP were associated with increased expression of proteins regulating metabolic pathways, including lipin-phosphatidic acid signalling, while in HHTg rats, beetroot administration was associated with reduced expression of proteins involved in mTOR signalling, inflammation, and cytoskeleton rearrangement.

### 4.1. Increased Hepatic Expression of Enzymes Regulating Glycerophospholipid Metabolism in SHR-CRP Rats after Treatment with Beetroot

Phosphatidic acid is a key intermediate metabolite in the synthesis pathways of all membrane glycerophospholipids, including phosphatidylcholine (PC), phosphatidylethanolamine (PE), phosphatidylserine (PS), and phosphatidylinositol (PI). Aside from this contribution to membrane biogenesis, phosphatidic acid is an important signalling lipid [30]. Several proteins involved in phosphatic acid metabolism were upregulated in SHR-CRP rats after treatment with beetroot. Some of these proteins were associated with lipid accumulation in the liver in animal models.

The AGPAT2 (1-acylglycerol-3-phosphate-O-acyltransferase 2) protein, which is located within the endoplasmic reticulum membrane, converts lysophosphatidic acid to phosphatidic acid as the second step in de novo phospholipid biosynthesis. It was reported that mice with a targeted mutation in the *Agpat2* gene developed severe lipodystrophy, affecting both white and brown adipose tissue and causing extreme insulin resistance, diabetes, and hepatic steatosis [31].

Phosphatidic acid is dephosphorylated by phosphatidate phosphatase LIPIN 1 and thus converted to diacylglycerol (DAG) and cytidine diphosphate diacylglycerol (CDP-DAG). DAG is the substrate for synthesis of other abundant phospholipids, such as phosphatidylcholine and phosphatidylethanolamine, while CDP-DAG is used to make several phospholipids, including phosphatidylglycerol and phosphatidylinositol. Mice with spontaneous mutations, including the fatty liver dystrophy (*fld*) gene that was later identified as the *Lpin1* gene, exhibit loss of body fat, fatty liver, hypertriglyceridemia, and insulin resistance [32]. Aside from lipid synthesis, LIPIN also acts as a transcriptional coactivator, interacting with PGC-1α, PPARα, and other factors that promote fatty acid oxidation [33].

The CEPT1 (choline-enthanolamine phosphotransferase 1) enzyme catalyses biosynthesis of both phosphatidylcholine (PC) and phosphatidylethanolamine (PE) from CDP-choline and CDP-ethanolamine, respectively. Changes in the PC and/or PE content of various tissues were implicated in metabolic disorders such as atherosclerosis, insulin resistance, and obesity [34].

SPTLC2 (serine palmitoyltransferase long chain base subunit 2) is a subunit of serine palmitoyltransferase (SPT). The SPT complex is the key enzyme in sphingolipid biosynthesis. SPT catalyses the rate-limiting step in the de novo ceramide biosynthesis pathway. In mice with adenovirus-induced overexpression of the *Sptlc2* gene in the liver, hepatic ceramide was found to modulate hepatosteatosis and the insulin response via increased VLDL secretion and inhibition of gluconeogenesis in vivo [35].

The PEMT (phosphatidylethanolamine N-methyltransferase) enzyme converts phosphatidylethanolamine (PE) to phosphatidylcholine (PC) via three sequential methylations by S-adenosylmethionine (SAM) in the liver. Hepatic PEMT is critical for maintaining phospholipid balance. It has been reported that *Pemt^-/-^* knockout mice that were fed a high-fat diet were protected against diet-induced obesity and insulin resistance, but developed non-alcoholic fatty liver disease (NAFLD) associated with a decreased PC:PE ratio [36].

The ETNK2 (ethanolamine kinase 2) enzyme catalyses the first step in PE biosynthesis via the CDP-ethanolamine pathway. Surprisingly, mice with targeted *Etnk2* genes showed no differences in hepatic PE when compared to wild-type controls [37].

The PNPLA7 (patatin-like phospholipase domain containing 7) enzyme functions as a lysophosphatidylcholine (LPC) hydrolase. It plays a critical role in regulating hepatic VLDL secretion by modulating ApoE stability through its interaction with ApoE. Hepatic TAG levels in *Pnpla7* knockdown mice were significantly higher than those of the control mice. In contrast, total cholesterol and FFAs in the liver either remained unchanged or only increased slightly. On the other hand, fasting plasma TAG in PNPLA7-knockdown mice were reduced when compared with the control mice [38].

### 4.2. Increased Hepatic Expression of Enzymes Regulating S-Adenosylmethionine (SAM) Biosynthesis in SHR-CRP Rats after Treatment with Beetroot

Disturbed S-adenosylmethionine (SAM) homeostasis in the liver is associated with hepatic steatosis in mice [39]. As mentioned above, the PEMT enzyme that converts PE to PC in the liver needs SAM for this reaction. MAT2 (methionine adenosyltransferase 2A) enzyme converts methionine to SAM; it is the only SAM synthetase expressed in most cells.

### 4.3. Increased Hepatic Expression of Enzymes Regulating Nicotinamide Adenine Dinucleotide (NAD^+^) Metabolism in SHR-CRP Rats after Treatment with Beetroot

The NAPRT (nicotinate phosphoribosyltransferase) enzyme transforms nicotinic acid (NA) to nicotinic acid mononucleotide (NAMN), which is then conjugated with ATP to generate NAD. NAD^+^ supplementation with NAD^+^ precursors ameliorate fatty liver disease [40].

The NNMT (nicotinamide N-methyltransferase) enzyme catalyses the reaction between nicotinamide (NAM) and SAM to produce 1-methylnicotinamide and S-adenosylhomocysteine. Recently, this enzyme has also been reported to modulate hepatic nutrient metabolism. Transgenic mice overexpressing NNMT elucidated its role in hepatic nutrient metabolism. When fed a high-fat diet containing NAM, a precursor for NAD^+^, these NNMT-overexpressing mice exhibited fatty liver deterioration, following increased expression of the genes mediating fatty acid uptake as well as decreased very low-density lipoprotein secretion [41].

### 4.4. Increased Hepatic Expression of Cytochromes P450, Enzymes Regulating Glutathiome Metabolism and Retinol Metabolism in SHR-CRP Rats after Treatment with Beetroot

Additional proteins with increased hepatic expression in SHR-CRP rats after beetroot treatment were identified. These include cytochrome P450 proteins. A mouse model where hepatic P450 activity has been reduced by >95% by the conditional deletion of the *Por* (P450 oxidoreductase) gene showed hepatic steatosis. These data suggest that the P450 system is a key regulator of hepatic lipid homoeostasis and liver growth [42].

Increased hepatic expression of the enzymes involved in glutathione metabolism in SHR-CRP rats treated with beetroot might contribute to reduced oxidative stress in the liver. Genes encoding the synthesis and activity of the glutathione S-transferase (GST) enzyme family are involved in the development of non-alcoholic fatty liver disease (NAFLD). GSTs possess protective activity against endogenous oxidative stress, as well as exogenous toxins, catalysing conjugation of the sulfhydryl groups of reduced glutathione and neutralising lipid oxidation products [43].

Reduced oxidative stress in SHR-CRP rats treated with beetroot was associated with increased expression of enzymes from the retinal biosynthetic pathway. ADH1 and ADH4 enzymes converted retinol to retinoic acid. Studies on mice with targeted *Adh1* and *Adh4* genes indicated that ADH1 provides considerable protection against vitamin A toxicity, whereas ADH4 promotes survival during vitamin A deficiency [44].

### 4.5. Reduced Expression of Proteins Involved in mTOR Signalling in HHTg Rats Treated with Beetroot

The AKT serine/threonine kinase (coded by *Akt2* gene) is an upstream positive regulator of mTOR. AKT activates mTOR via direct phosphorylation and inhibition of tuberous sclerosis complex 2 (TSC2), which is a negative regulator of mTOR. The marked hyperglycaemia and loss of pancreatic β cells and adipose tissue in *Akt2*-deficient mice suggest that AKT plays critical roles in glucose metabolism and the development or maintenance of proper adipose tissue and islet mass [45].

The *Mtor* (mechanistic target of rapamycin kinase) gene codes for a protein that belongs to a family of phosphatidylinositol kinase-related kinases. These kinases mediate cellular responses to stresses such as DNA damage and nutrient deprivation. This kinase is a component of two distinct complexes: mTORC1, which controls protein synthesis, cell growth, and proliferation; and mTORC2, which is a regulator of the actin cytoskeleton and promotes cell survival and cell cycle progression. mTOR is a central regulator of lipid metabolism, regulating not only lipogenesis and lipolysis, but also adipogenesis. mTORC1 drives lipid synthesis through the transcription factors sterol regulatory element binding protein 1/2 (SREBP1/2) and peroxisome proliferator-activated receptor-γ (PPARγ). When sterol levels are low, the SREBPs translocate from the endoplasmic reticulum membrane to the nucleus, where they upregulate genes for de novo lipid and cholesterol synthesis. Activated mTORC1 promotes this SREBP transcriptional programme by phosphorylating the SREBP inhibitor LIPIN 1 to exclude it from the nucleus [46,47].

The *Deptor* (DEP domain-containing mTOR-interacting protein) gene codes for a protein that is involved in the mTOR signalling pathway as an endogenous regulator. Liver-specific *Deptor* null mice showed a reduction in circulating glucose and reduced hepatic glycogen content, as well as sustained mTORC1 activation, when fasted [48].

The *Rictor* (RPTOR independent companion of MTOR complex 2) gene codes for a protein that is specific for mTORC2. RICTOR and mTORC2 have been shown to play an essential role in embryonic growth and development, perhaps due to the control that mTORC2 exerts on actin cytoskeleton organisation. Mice lacking the *Rictor* gene in the liver were unable to respond normally to insulin when they failed to inhibit hepatic glucose output. KO mice also failed to develop hepatic steatosis on a high-fat diet and manifested half-normal serum cholesterol levels [49].

The *Lpin3* (lipin 3) gene codes for an enzyme that catalyses dephosphorylation of phosphatidate to diacylglycerol. Lipins also possess a transcriptional coactivator motif and have been shown to co-regulate the expression of fatty acid oxidation genes. Lipin-1 and lipin-3 cooperate in vivo to determine adipose tissue PAP activity and adiposity [50].

### 4.6. Reduced Expression of Proteins Involved in Cellular Trafficking in HHTg Rats Treated with Beetroot

Lipid synthesis and lipid droplet formation, as well as lipid mobilisation and degradation, involve multiple cellular compartments. Therefore, to maintain hepatocellular lipid homeostasis and to coordinate lipid synthesis, storage, degradation, and secretion, cellular organelles have to dynamically interact.

The *Klc1* (kinesin light chain 1) gene codes for a protein that is a subunit of kinesin 1. Kinesin-1 binds to phophatidic acid on lipid droplets, transporting them to the periphery of hepatocytes, where contacts form between the lipid droplets and the ER [51].

The *Dctn6* (dynactin subunit 6) gene codes for a protein that is a part of a dynactin complex. Dynactin is a 23-subunit protein complex that acts as a co-factor for the microtubule motor cytoplasmic dynein-1. Dyneins are a family of cytoskeletal motor proteins that move along microtubules in cells [52].

The *Actr1b* (actin related protein 1B) gene codes for a subunit of dynactin.

The *Exoc4* and *Exoc5* (exocyst complex component 4 and 5) genes code for proteins that are components of the exocyst complex, a multiple protein complex essential for targeting exocytic vesicles to specific docking sites on the plasma membrane. Components of the exocyst complex interact with the actin cytoskeletal remodelling and vesicle transport machinery. The exocyst complex plays an important role in the regulation of FFA uptake by adipocytes [53].

The *Tuba1a* and *Tuba3a* (tubulin alpha 1a and 3a) genes code for proteins that are parts of microtubules of the eukaryotic cytoskeleton. Tubulin polymerisation was also found to regulate FFA uptake by adipocytes in collaboration with the exocyst complex.

The *S100a10* (S100 calcium-binding protein A10) gene codes for a protein linked to vesicle trafficking.

The *Pik3c3* (phosphatidylinositol 3-kinase catalytic subunit type 3) gene codes for a protein that is involved in endosome transport and regulation of cytokinesis. PIK3C3 plays important roles in intracellular membrane trafficking.

### 4.7. Reduced Expression of Proteins Involved in Inflammation and Apoptosis in HHTg Rats Treated with Beetroot

The *Chuk* (component of inhibitor of nuclear factor kappa B kinase complex) gene codes for a protein that belongs to inhibitory kappa kinases (IKK), which are involved in the activation of NF-κB, a master regulator of inflammatory response. In humans, CHUK polymorphisms are associated with hepatic steatosis [54].

The *Ikbkg* (inhibitor of nuclear factor kappa B kinase regulatory subunit gamma) gene codes for a protein (NEMO) which activates NF-κB, resulting in the activation of genes involved in inflammation, immunity, cell survival, and other pathways.

The *Irak1* (interleukin 1 receptor associated kinase 1) gene is partially responsible for IL1-induced upregulation of the transcription factor NF-κB. Toll-like receptors (TLRs) detect pathogens, and the IL-1 receptor (IL-1R) family enables cells to quickly respond to inflammatory cytokines by mounting an efficient protective response. Interleukin-1 receptor activated kinases (IRAKs) are key mediators in the signalling pathways of TLRs/IL-1Rs.

### 4.8. Molecular Mechanisms of Beetroot Effects on Oxidative Stress, Inflammation, and Metabolism

It has been reported that treatment of Wistar rats with type 2 diabetes induced by STZ with methanolic beetroot extract and a high-fat diet reduced serum and hepatic levels of cholesterol, triglycerides, and free fatty acids, which was associated with downregulated transcription of SREBP1/2 and stimulation of PPARα [55]. Congruent with these results, we observed that the hypolipidemic effects of beetroot in SHR-CRP rats were associated with increased fatty acid oxidation in the skeletal muscle.

Betanin treatment of rats with STZ-induced type 2 diabetes was associated with amelioration of insulin resistance and improved lipid profile, accompanied with modulation of the AMPK/SIRT1/NF-κB signalling pathway [56].

Beetroot extracts were shown to reduce expression of mTOR signalling in a HeLa cervical cancer cell line [57].

## 5. Conclusions

Beetroot treatment reduced oxidative stress, weight of visceral fat, and ectopic fat accumulation in SHR-CRP rats without affecting the levels of inflammatory markers or the expression of proteins from inflammatory pathways. Amelioration of lipid metabolism was associated with increased expression of proteins involved in lipin-phosphatidic acid signalling. Similar antioxidative and hypolipidemic effects of beetroot treatment in HHTg rats were associated with reduced expression of the proteins involved in mTOR signalling. Despite the fact that protein profiling experiments are of a correlational or descriptive nature, our studies generated new hypotheses for more powerful and focused strategies to untangle the complex web of causal relationships in follow-up experiments.

## Figures and Tables

**Table 1:** Parameters of glucose and lipid metabolism in SHR-CRP and HHTg rats treated with beetroot juice and in their untreated controls. ND denotes not determined. * denotes *p* < 0.05.

Trait	SHR-CRP Control	SHR-CRP Beetroot	HHTG Control	HHTG Beetroot
Body weight (g)	315 ± 6	306 ± 8	426 ± 6	427 ± 9
Serum cholesterol (mmol/L)	1.45 ± 0.06	1.47 ± 0.04	1.63 ± 0.10	1.58 ± 0.06
Serum HDL cholesterol (mmol/L)	1.22 ± 0.04	1.28 ± 0.02	0.785 ± 0.062	0.848 ± 0.039
Non-fasting glucose (mmol/L)	7.06 ± 0.10	6.95 ± 0.17	9.38 ± 0.41	9.09 ± 0.32
Serum insulin (nmol/L)	0.247 ± 0.011	0.237 ± 0.030	0.178 ± 0.022	0.219 ± 0.029
M. gastrocnemius triglycerides (μmol/g)	2.03 ± 0.21	1.98 ± 0.62	3.92 ± 0.67	3.70 ± 0.41
Heart triglycerides (μmol/g)	1.91 ± 0.07	1.40 ± 0.16 *	3.52 ± 0.45	3.55 ± 0.38
Basal lipogenesis (nmol gL./g/2 h)	1009 ± 79	1726 ± 332	210 ± 15	230 ± 15
Stimulated lipogenesis (nmol gL./g/2 h)	1436 ± 148	2339 ± 507	241 ± 16	239 ± 20
Basal glycogenesis (nmol gL./g/2 h)	996 ± 182	1089 ± 174	ND	ND
Stimulated glycogenesis (nmol gL./g/2 h)	1548 ± 175	1358 ± 241	ND	ND
Plasma cGMP (pmol/L)	8.84 ± 0.64	13.41 ± 1.22 *	ND	ND
cGMP in aorta (pmol/g)	40.66 ± 8.88	59.35 ± 27.57	ND	ND
cGMP in myocardium (pmol/g)	12.25 ± 1.08	23.37 ± 1.44 *	ND	ND

**Table 2:** Parameters of inflammation in serum.

Trait	SHR-CRP Control	SHR-CRP Beetroot	HHTg Control	HHTg Beetroot
rat hsCRP (mg/mL)	0.375 ± 0.018	0.329 ± 0.039	0.675 ± 0.030	0.729 ± 0.049
human CRP (mg/L)	163 ± 6	184 ± 19	u.d.	u.d.
MCP-1 (pg/mL)	6.96 ± 0.30	5.81 ± 0.36	3.29 ± 0.21	3.68 ± 0.16
IL6 (pg/mL)	39.13 ± 7.36	20.83 ± 6.17	44.27 ± 10.12	48.35 ± 13.79
leptin (ng/mL)	3.07 ± 0.22	5.17 ± 0.67	13.80 ± 1.00	11.56 ± 1.13
TNFα (pg/mL)	4.97 ± 0.32	4.76 ± 0.38	5.58 ± 0.53	6.36 ± 0.38

**Table 3:** Oxidative stress parameters in the liver.

Trait	SHR-CRP Control	SHR-CRP Beetroot	HHTg Control	HHTg Beetroot
SOD (U/mg protein)	0.124 ± 0.011	0.127 ± 0.007	0.111 ± 0.005	0.130 ± 0.004 *
GSH-Px (μM NADPH/min/mg protein)	271 ± 17	300 ± 22	211 ± 36	419 ± 48 **
GR (μM NADPH/min/mg protein)	123 ± 9	153 ± 15	73 ± 7	85 ± 5
CAT (mM H_2_O_2_/min/mg protein)	1603 ± 188	2274 ± 94 *	1365 ± 23	1500 ± 50 *
GSH (μmol/mg protein)	52.09 ± 3.60	58.09 ± 1.88	61.14 ± 1.62	61.12 ± 3.43
GSSG(μmol/mg protein)	4.08 ± 0.44	4.42 ± 0.45	3.01 ± 0.22	2.80 ± 0.19
GSH/GSSG	12.96 ± 0.41	13.33 ± 0.97	20.92 ± 1.83	22.33 ± 1.74
Conjugated dienes (nM/mg protein)	41.5 ± 3.3	30.2 ± 2.3 *		
TBARS/MDA (nM/mg protein)	1.73 ± 0.17	1.63 ± 0.13	1.82 ± 0.11	1.89 ± 0.12

* and ** denote *p* < 0.05 and *p* < 0.005, respectively.

**Table 4:** Genes from significant KEGG pathways coding for differential hepatic expression of proteins in SHR-CRP rats treated with beetroot juice versus SHR-CRP untreated controls.

KEGG Pathway	Counts	*p*-Value	Benjamini
Metabolic pathways	65	3.0 × 10^−19^	6.2 × 10^−17^
↑*Aox1*, ↑*Aox3*, ↑*Akr1d1*, ↑*Amdhd1*, ↑*Aoc3*, ↑*Car14*, ↑*Cept1*, ↑*Cyp2a1*, ↑*Cyp2a*2, ↑*Cyp2c13*, ↑*Cyp2c7*, ↑*Cyp4a2*, ↑*Cox6a1*, ↑*Cox6b1*, ↑*Dpys*, ↑*Ephx2*, ↑*Etnk2*, ↑*Flad1*, ↑*Fmo5*, ↑*Fcsk*, ↑*Ggt5*, ↑*Gne*, ↑*Gsta2*, ↑*Gsta5*, ↑*Gstm3*, ↑*Gstm4*, ↑*Hykk*, ↓*Hsd17b2*, ↓*Ido2*, ↓*Inmt*, ↓*Impa2*, ↑*Lpin1*, ↑*Mat2a*, ↓*Mocs2*, ↑*Nnmt*, ↑*Naprt*, ↑*Pemt*, ↑*Pde8a*, ↓*Plod1*, ↑*Prodh2*, ↑*Rdh5*, ↓*Sptlc2*, ↑*Srd5a1*, ↑*Sdhd*, ↑*Tat*, ↑*Urad*, ↑*Vnn3*			
Retinol metabolism	14	7.1 × 10^−11^	7.4 × 10^−9^
↑*Ugt1a7*, ↑*Adh1*, ↑*Adh4*, ↑*Aldh1a1*, ↑*Aldh1a7*, ↑*Aox1*, ↑*Aox3*, ↑*Cyp2a1*, ↑*Cyp2a2*, ↑*Cyp2c13*, ↑*Cyp2c7*, ↑*Cyp4a2*			
Steroid hormone biosynthesis	8	1.0 × 10^−4^	3.6 × 10^−3^
↑*Ugt1a7*, ↑*Akr1d1*, ↑*Cyp2c13*, ↑*Cyp2c7*, ↑*Cyp2d3*, ↓*Hsd17b2*, ↑*Srd5a1*			
Tyrosine metabolism	6	1.3 × 10^−4^	3.8 × 10^−3^
↑*Adh1*, ↑*Adh4*, ↑*Aox1*, ↑*Aox3*, ↑*Aoc3*, ↑*Tat*			
Glycerophospholipid metabolism	7	1.8 × 10^−3^	4.2 × 10^−2^
↑*Agpat2*, ↑*Cept1*, ↑*Lpin1*, ↑*Pnpla6*, ↓*Pnpla7*, ↑*Pemt*			
Glutathione metabolism	6	2.4 × 10^−3^	4.9 × 10^−2^
↑*Ggt5*, ↑*Gsta2*, ↑*Gsta*5, ↑*Gstm3*, ↑*Gstm4*			

↑ denotes upregulated hepatic protein expression in SHR-CRP rats treated with beetroot versus untreated controls; ↓ denotes downregulated hepatic protein expression in SHR-CRP rats treated with beetroot versus untreated controls.

**Table 5:** Genes from significant KEGG pathways coding for differential hepatic expression of proteins in HTTg rats treated with beetroot juice versus HTTg untreated controls.

KEGG Pathway	Counts	*p*-Value	Benjamini
mTOR signalling	12	2.3 × 10^−4^	4.7 × 10^−2^
↓*Akt2*, ↓*Braf*, ↓*Clip1*, ↓*Deptor*, ↓*Rictor*, ↓*Raf1*, ↓*Stradb*, ↓*Chuk*, ↓*Lamtor3*, ↓*Lpin3*, ↓*Mtor*, ↓*Pik3r1*			
Salmonella infection	15	3.9 × 10^−4^	4.7 × 10^−2^
↓*Akt2*, ↓*Raf1*, ↓*S100a10*, ↓*Actr1b*, ↓*Casp7*, ↓*Chuk*, ↓*Dctn6*, ↓*Exoc4*, ↓*Exoc5*, ↓*Ikbkg*, ↓*Irak1*, ↓*Klc1*, ↓*Pik3c3*, ↓*Tuba1a*, ↓*Tuba3a*			
Alzheimer disease	19	5.3 × 10^−4^	4.7 × 10^−2^
↓*Akt2*, ↓*Braf*, ↓*Ndufa1*, ↓*Ndufv3*, ↓*Raf1*, ↓*Casp7*, ↓*Chuk*, ↓*Cdk5*, ↓*Cox5a*, ↓*Ikbkg*, ↓*Klc1*, ↓*Mtor*, ↓*Mme*, ↓*Pik3c3*, ↓*Pik3r1*, ↓*Plcb1*, ↓*Slc39a4*, ↓*Tuba1a*, ↓*Tuba3a*			

↑ denotes upregulated hepatic protein expression in HHTg rats treated with beetroot versus untreated controls; ↓ denotes downregulated hepatic protein expression in HHTg rats treated with beetroot versus untreated controls.

## Data Availability

All data generated or analysed during this study are included in this published article (and its Supplementary Information files).

## Supplementary Materials

The following supporting information can be downloaded at: https://www.mdpi.com/article/10.3390/metabo13020192/s1, Table S1: Differential expression of hepatic proteins in SHR-CRP and HHTg rats treated with beetroot versus their untreated controls.
